# Health-Related Quality of Life and Family Functioning of Primary Caregivers of Children with Cerebral Palsy in Malaysia

**DOI:** 10.3390/ijerph18052351

**Published:** 2021-02-28

**Authors:** Kelvin Ying, Hans Van Rostenberghe, Garry Kuan, Mohammad Haris Amirul Mohd Yusoff, Siti Hawa Ali, Nik Soriani Yaacob

**Affiliations:** 1 Interdisciplinary Health Sciences Unit, School of Health Sciences, Universiti Sains Malaysia, Kubang Kerian 16150, Kelantan, Malaysia; klvnying@gmail.com; 2 Cerebral Palsy Research Cluster, Universiti Sains Malaysia, Kubang Kerian 16150, Kelantan, Malaysia; hansvro@usm.my (H.V.R.); garry@usm.my (G.K.); harisamirul@usm.my (M.H.A.M.Y.); 3 Department of Paediatrics, School of Medical Sciences, Universiti Sains Malaysia, Kubang Kerian 16150, Kelantan, Malaysia; 4 Exercise and Sports Science, School of Health Sciences, Universiti Sains Malaysia, Kubang Kerian 16150, Kelantan, Malaysia; 5 Department of Chemical Pathology, School of Medical Sciences, Universiti Sains Malaysia, Kubang Kerian 16150, Kelantan, Malaysia

**Keywords:** disability, cerebral palsy, caregiver, HRQOL, family functioning

## Abstract

Caregiving for children with cerebral palsy (CP) has proved to negatively impact on the physical and psychological well-being of their primary caregivers. The aim of the current study was to examine the overall impact of caregiving for children with CP on the primary caregivers’ health-related quality of life (HRQOL) and family functioning, and to identify potential factors associated with primary caregivers’ HRQOL and family functioning. The cross-sectional study involved a total of 159 primary caregivers of children with CP with a mean age of 42.8 ± 8.4 years. Demographic data and information on the physical and leisure activities of the primary caregivers were collected, and their quality of life (QOL) was measured based on the self-reported Pediatric Quality of Life Inventory Family Impact Module (PedsQL FIM). Primary caregivers in the current study have shown good HRQOL and family functioning, with scores of 82.4 and 85.3 out of 100, respectively. Through multiple linear regression analyses, the mother’s level of education, family monthly income, sleeping problems in children with CP, and the existence of children with other types of disability have been identified as factors contributing to HRQOL and family functioning. The findings help set out the course for stakeholders to establish action to enhance the QOL of primary caregivers.

## 1. Introduction

It is estimated that there are about 460,000 children with disability (CWD) in Malaysia. This is based on the 5% prevalence of 9.3 million children among the Malaysian population [1,2,3]. The Department of Social Welfare (DSW) Malaysia registered 453,258 persons with disability (both adults and children) in 2017 [1]. DSW registration is based on seven specific categories which are physical disability, visual disability, hearing disability, speech disability, learning disability, mental disability, or ‘other’ disabilities (that include multiple disabilities, or other categories not mentioned in the earlier grouping) [4]. Children with cerebral palsy (CP) are categorized under physical disability and thus identifying them becomes very difficult. Nevertheless, the cumulative number of registered children with CP between 2011 and 2017 was 5840 (direct communication with DSW). The Ministry of Health Malaysia on the other hand, registered 2766 children with special needs in 2012, of whom 215 were children with CP [5]. However, based on the estimated prevalence of 2 to 2.5 CP per 1000 live births in the developed countries [6,7,8,9], the actual number of children with CP in Malaysia may be significantly higher. The lack of specific data on children with CP proves difficult not only in supporting research evidence but most importantly in planning delivery of services that can provide better support for both the children and their primary caregivers.

The burden on families of children with CP begins with the confirmation and disclosure of the diagnosis. Indeed, an Australian study reported the feeling of sadness in 98% of primary caregivers and that chronic sorrow symptoms continued to persist even years after diagnosis [10]. Subsequently, their life needs to be adapted to enable them to provide optimal care for the children with CP and to adapt emotionally to an unexpected situation. The permanence of the disability imposes continuous and escalating burden on the children and their families. There is growing evidence that caring for children with special needs negatively impacts primary caregivers by causing sadness, feeling of overwhelming, anxiety, depression, worry, low physical health, bad family functioning and low quality of life (QOL) compared to families with healthy children [11,12,13]. This multifactorial impact on the primary caregivers is long-term and could in turn affect the quality of parenting or primary caregiver-child relationship. As children with CP are prone to have behavioral problems [14,15], differentiating between CP-associated responses and behavioral issues is challenging for primary caregivers [14], which may further impact on their coping strategies. Primary caregivers of children with CP have five times higher risk of developing clinical depression compared to normal primary caregivers [16].

Although CP is a global phenomenon, the impact of parenting children with CP in Malaysia may differ from other countries, considering differences in the health care system, culture, socio-economic status, and educational background of the primary caregivers. It is crucial that factors affecting the well-being of Malaysian primary caregivers and their families are identified to help them adequately cope with managing children with CP [11,17] without significantly compromising their own QOL. As such, the current study (1) determined the overall impact of having a child with CP on the health-related quality of life (HRQOL) of the primary caregivers, and family functioning; (2) identified potential factors which may affect the primary caregivers’ HRQOL and family functioning.

## 2. Materials and Methods

The study reported herein was part of our larger cross-sectional study supported by community engagement through the Key Informant Method (KIM). KIM was developed by the International Centre for Evidence in Disability at the London School of Hygiene and Tropical Medicine (together with CBM, formerly known as Christian Blind Mission) as an approach to identify CWD in the community through trained key informants (KIs). The larger study was conducted through organizing health screening camps in three states in Malaysia.

### 2.1. Participants

A total of 444 CWDs (of which 172 are children with CP) and their primary caregivers attended the health screening camps conducted in Kelantan, Johor and Sarawak states. For the current study, the target population was primary caregivers of children with CP. G-power software was used for statistical power analysis to determine the study sample size for level of significance 0.05, effect size 0.15 (medium) [18] and power 0.80.The required minimum sample size was calculated to be 169. The total number of respondents for this study was 172. However, some responses to the questionnaires were incomplete and the data were therefore excluded. Thus, the final analyses were performed on 159 complete sets of data, which are adequate for inferential analysis using multiple linear regression. The ethnicity of the final 159 participants was Malay (76.1%), Chinese (11.3%), Indian (5.7%), Ibans (5.0%) and others (1.9%). All participants were Malaysian and literate in Malay language for reading, speaking and writing.

### 2.2. Data Collection

The study received ethical approval from the Universiti Sains Malaysia (USM) Human Research Ethics Committee (USM/JEPeM/16100411) and was conducted in accordance with the guidelines of the International Declaration of Helsinki. KIM was employed which involved a two-stage identification and assessment process for children with disability. The KIs included representatives of local governmental and non-governmental organizations (NGOs) and community leaders. They were members of the public who lived within the vicinity of the project areas and provided services to the community. These included village heads, religious representatives, nurses from the community clinics, social workers from district offices, care workers from community rehabilitation centers and workers/volunteers from NGOs. KIs were first explained about the study and then invited to two half-day small group training sessions. The training provided more information on the study, including methodological approaches adopted. The KIs were trained to identify children with physical disabilities at the community level. Information regarding services to be provided during the health screening camps and ethical practices such as maintaining confidentiality of information, was also shared. Pre-and post-training questionnaires were provided to examine the KIs’ level of understanding regarding CP and other forms of disability. Through these KIs, CWDs and their primary caregivers in the selected areas were invited to attend the health screening camps and during this period, the research team members were in continuous contact with the KIs. Identified children attended health screening camps in the urban areas of Kelantan, Johor or Sarawak (December 2016–August 2018) where they received free medical and health assessments conducted by trained health and medical officers as well as specialists. Most KIs also attended the health screening camps as a support to the primary caregivers. They were also invited to the feedback sessions conducted a few months after completion of each health screening camp.

At the health screening camp, primary caregivers were provided with one portfolio that consisted of a list of assessments available for their children and one set of questionnaires. The enumerators assisted in the filling of the questionnaires that were related to their sociodemographic status and the clinical history of their children. The children were then invited to complete all the medical and health examination stations that included pediatrics, ophthalmology, ENT, oral health, occupational therapy, physiotherapy speech and nutritional status analysis. As a follow up, the attending specialist would issue a referral letter for further investigation at a nearby health care facility, if necessary. The entire assessment lasted approximately 30–45 min.

Together with the medical record, the questionnaires consisted of sociodemographic questions and the Malay version of the Pediatric Quality of Life Inventory (PedsQL), including the Family Impact Module (FIM). Potential participants (primary caregivers) were briefed regarding the study. Primary caregivers who volunteered to participate in the study and were able to give informed consent were surveyed.

### 2.3. Instruments

#### 2.3.1. Demographic/Physical and Leisure Activities

Several sociodemographic questions were asked in the questionnaire. These questions assessed the personal attributes of primary caregivers and their children with CP, including age, biological sex and physical condition (i.e., types of CP, Gross Motor Function Classification System (GMFCS) level and other comorbidities) of the child, residing area, primary caregivers’ education level, family monthly income, existence of other CWDs in the family, child attendance to school and access to healthcare services.

#### 2.3.2. Pediatric Quality of Life Inventory^TM^ Family Impact Module (PedsQL FIM)

The study investigated the impact of chronic health conditions of the children with CP on primary caregivers and their families by applying PedsQL FIM. The original PedsQL FIM was validated among 23 families of children with complex chronic health conditions in San Diego by Varni et al. [19] with good internal consistency reliability (α = 0.82 to 0.97), while the Malay version of PedsQL FIM was validated among 383 respondents from the state of Kelantan by Isa et al. [20] with Cronbach’s alpha ranging from 0.75 to 0.94.

PedsQL FIM is a parent reported scale consisting of 36 items with eight subscales, six of which measure parent self-functioning (i.e., physical functioning, emotional functioning, cognitive functioning, social functioning, communication and worry) and two other subscales that measure family functioning (i.e., daily activities and family relationships). The 5-point Likert scale FIM was rated with 0 = never a problem, 1 = almost never a problem, 2 = sometimes a problem, 3 = often a problem, and 4 = almost always a problem. Items were reversed scored and linearly transformed to a 0 to 100 scale (0 = 100, 1 = 75, 2 = 50, 3 = 25, and 4 = 0). Higher scores on this scale indicate better functioning (less negative impact). The Total Impact Score was measured as the sum of all 36 items divided by the number of items answered. The Parent HRQOL Summary Scores were computed as the sum of the items divided by the number of items answered in the physical, emotional, social and cognitive functioning scales. The Family Functioning Summary Scores were computed as the sum of the items divided by the number of items answered in the daily activities and family relationships scale.

### 2.4. Statistical Analysis

All statistical analyses were performed using SPSS version 27.0 (IBM Corp., Armonk, NY, USA). Data on the profile of primary caregivers and their children with CP, and each domain of PedsQL FIM (responded by primary caregivers) were analyzed using descriptive statistics. The relationship between each predictor variable and outcome variables (i.e., Total Impact Score, Parent HRQOL Summary Score and Family Functioning Summary Score) was investigated using simple linear regression. Finally, all significant predictor variables were then included in a multiple linear regression model. A *p* value of 0.05 was set as the cut-off for statistical significance. Interactions, multicollinearity, model fitness and assumptions, outliers and influential cases were checked. Regression coefficients (b), 95% confidence intervals (CI), *p*-values, and overall coefficient of determination (R2) values were presented in the final model.

## 3. Results

### 3.1. The Profile of Primary Caregivers and Their Children with CP

Table 1 shows the profile of the primary caregivers who participated in this study. The total number of primary caregiver participants was 159. The distribution of participants was as follows: 67.9% from Johor, 22.0% from Kelantan and 27.0% from Sarawak health screening camps. The age of primary caregivers ranged from 21–64 years old (M = 42.8, SD = 8.4). Majority of the primary caregivers were female (79.9%), with family income of less than RM2000 (~USD494) (43.4%) and do not parent or care for any other CWD (88.7%). Majority of them (child’s father, 84.3%; child’s mother, 84.9%) completed at least secondary education.

The profiles of the children with CP are shown in Table 2. Nearly 60% of the children were male and are 8-18 years of age (67.9%). More than half of the children with CP have access to healthcare services, such as rehabilitation centers, hospitals and clinics. Nearly half of them, however, do not attend any mainstream or special school. Most of these children had spastic CP (78.0%) with more than half of them classified as level IV (21.4%) and level V (38.4%) in GMFCS. The top three most reported comorbidities were speech problems (66.0%), learning disabilities (56.6%) and bowel problems (50.9%), while the three least reported comorbidities were hearing problems (8.2%), gastroesophageal reflux disease (6.9%) and hyperactive airway disease (2.5%). The mean number of comorbidities experienced by the children with CP is 4.0 (3.0).

Table 3 shows the mean scores of PedsQL FIM according to each subscale, primary caregivers HRQOL, family functioning and total family impact. Higher scores indicate better primary caregiver HRQOL and family functioning. The mean (SD) of Total Impact Score, Parent HRQOL Summary Score and Family Functioning Summary Score were 81.9 (12.90), 82.4 (13.64) and 85.3 (14.69), respectively. The 3 highest subscale scores were the mean score for “family relationship” (M = 90.9, SD = 13.81), “social functioning” (M = 84.6, SD = 17.72) and “cognitive functioning” (M = 85.0, SD = 16.55). The lowest score was the mean score for “daily activities” (M = 76.1, SD = 23.84). The internal consistency reliability for each subscale, two summary scores and Total Impact Score of the FIM are also presented in Table 3. Most of them have Cronbach’s alpha coefficients higher than 0.70, except for “communication” and “worry”.

### 3.2. Predictor Variables Associated with Family Impact Module

#### 3.2.1. Total Impact Score

We have tested variables from Table 1 and Table 2 as predictors and only report those which show significant results. In Table 4, multiple linear regression (MLR) analysis showed that the mother’s level of education and family monthly income significantly predicts the total family impact. About 11% of the variations in the total impact score was explained by these variables (*R*^2^ = 0.110). After adjusting for other variables, the findings revealed that families with mothers who received education beyond secondary level (compared to those with no education/primary school level) had a better experience total impact score. Primary caregivers earning high family monthly income (as compared to those with low family monthly income) displayed lower total impact score.

#### 3.2.2. Parent HRQOL Summary Score

The same predictor variables for total impact score have also shown significant association with the Parent HRQOL Summary Score. In addition, the sleeping problems of children with CP were significantly associated with the primary caregiver’s HRQOL, as shown in Table 5. Approximately 8.6% of the variations in the Parent HRQOL Summary Score were explained by these variables (*R*^2^ = 0.086). After adjusting for other variables, the findings showed families with mothers who received education beyond secondary level (compared to those with no education/primary school level) experienced better HRQOL. Primary caregivers with high family monthly income (compared to primary caregivers with low family monthly income) had a lower HRQOL score. We also noted a lower score of HRQOL among primary caregivers of children with CP who were having sleeping problems.

#### 3.2.3. Family Functioning Summary Score

The predictor variables that are significantly associated with the Family Functioning Summary Score were the level of education of the mother, family monthly income and the presence of children with other disabilities in the family. Approximately 10.3% of the variations in this score were explained by these variables (*R*^2^ = 0.103). After adjusting for other variables, the findings revealed that parenting children with other disabilities contributes to better family functioning. Also, families with mothers who received education beyond secondary level (compared to those with no education/primary school level) experienced better family functioning. Primary caregivers who have high family monthly income (compared to primary caregivers with low family monthly income) had a lower Family Functioning Score, as shown in Table 6.

## 4. Discussion

The current study explores the sociodemographic profiles of Malaysian primary caregivers and their children with CP. The majority (75.5%) of the study respondents were mothers who spent most of their time taking care of their children with CP, as they had the most knowledge about their child’s condition. As they play the role of primary caregivers, they were able to provide detailed information on the condition of their children. Globally, many studies related to primary caregivers of children with CP have been conducted on mothers because involvement of other family members such as fathers, in caretaking, has always been lower [24,25,26,27]. Our current research has also shown a similar pattern whereby mothers made up most of the primary caregivers attending the health screening camps in all the three states involved.

Like other studies [28,29,30], most of the children with CP in the current study had spastic CP with the majority suffering from CP of GMFCS level IV and V (59.8%). It could be assumed that primary caregivers with more severe children with CP are more likely to take up the opportunity of services offered by health screening camp designed in this study, compared to those who have children with less severe CP. Nevertheless, statistical data on the different GMFCS levels of children with CP in the target communities were not available to proportionately estimate the CP groups attending the health screening camps. Access to facilities that enabled children with CP with GMFCS level IV and V to be transported to the health screening camps could be one of the reasons for their high attendance. As reflected by respondents in Pretorius and Steadman [31], primary caregivers are more willing to send their children with CP to attend healthcare services if they can overcome the transport-related barriers, including transportation fees, transports that are equipped to carry a wheelchair and poor road conditions.

The most reported comorbidities faced by children with CP in the current study were speech problems (66.0%), followed by learning disabilities (56.6%) and bowel problems (50.9%). Less than 10% of the respondents have reported their children with CP experiencing hearing problems, gastroesophageal reflux disease and hyperactive airway disease. This is similar to a study that investigated the motor function of pre-school children with CP in Bangladesh and Australia, with the most reported comorbidity being speech impairment, and the least reported hearing impairment [32].

Besides sociodemographic and CP-related factors, the current study also explored the HRQOL and family functioning of respondents. Results indicate that families in this study scored relatively higher primary caregiver’s HRQOL and family functioning compared to recent studies conducted in other countries such as Ghana, Poland, Bosnia and Herzegovina [33,34,35]. Families with children with CP in this study showed the highest score in family relationship. This indicates that at least in the Malaysian context, having a child with CP does not seem to affect the relationship between family members and communication within the family is going well.

Respondents in the current study scored the lowest mean score in overall daily activities but the value is not critically low (i.e., 76.1 out of 100). Respondents may have become familiar with CP care over the years, so they do not have a major problem in performing daily activities. This is in line with a previous study that reported the impact on the caregivers were more noticeable in the early years [36]. In the current study, 83.6% of the children are aged five to 18 years old, hence, certain level of adaptation may have probably been established. In addition, 99 out of 103 respondents reported having access to hospitals and clinics, while 89 out of 159 reported receiving support from rehabilitation centers. Adequate healthcare support system in Malaysia allows them to allocate more time to their household tasks. All these could perhaps explain our observation that most families of children with CP are still functioning well in their day-to-day activities.

The current data included the education level of both the father and mother of children with CP as possible predictors to total family impact, HRQOL, and family functioning. MLR analysis revealed that father’s education level does not have significant contribution to the three dependent variables. On the contrary, mother’s higher education level predicted higher Total Impact Scores, better HRQOL experiences and family functioning (compared to family with mothers who received no education/primary school level). Primary caregivers’ education level, which reflects their health literacy to some extent [37,38], plays an important role in their overall well-being. Primary caregivers with adequate health literacy have the capacity to gain, process, and comprehend any information and services needed for their own well-being, as well as for their children with CP, thus enabling self-care and care for their family. This finding as well as the fact that majority of primary caregivers attending the health screening camps are mothers, suggested the conventional social expectation for a mother in child caretaking, more than the father.

The effect of socio-economic status on the well-being of the children with CP’s family has always been ambiguous. A study by Bella and colleagues [36] with 37 mothers of children with CP found that perceived burden and stress were not influenced by income. On the other hand, by developing Structural Equation Modelling with data from primary caregivers of children with CP, Raina et al. [26] found that gross income was indirectly and positively associated with the physical and psychological health of the primary caregivers. Another study by Khayatzadeh et al. [39] in Tehran, which aimed to compare the QOL of mothers of children with CP with mothers of typically developing children, reported significant positive relationship between socio-economic status with all domains of WHOQOL-BREF, which are physical health, psychological health, social relationship, and environmental health. In comparison to primary caregivers with low monthly family income, the current study reported lower total impact score, HRQOL and family functioning of primary caregivers with high family monthly income. In addition, we also found that 15 out of 159 primary caregivers had children with other forms of disabilities as well. They indicated that this additional situation did not cause any issue in terms of family functioning.

Turning into religion might be one of the underlying reasons to such findings. Most Malaysians apply religious coping methods during times of difficulty. Research by Basri and Hashim [40], Isa et al. [13] and Mohamad et al. [41] revealed the role of religion in helping primary caregivers to cope with their physical and psychological health, while caring for their family members with disability or health issue. Primary caregivers of children with CP have emphasized on the need to stay positive and optimistic [10]. According to Haque [42], the holistic teaching of Islam in all areas of life includes the caregiving role by family members. This has positively impacted on the believers’ family-relative relationships. Religious beliefs also helped primary caregivers to accept their CWD, by viewing them as a gift from God and offering them sense of purpose [43]. Religious beliefs therefore help primary caregivers to develop more positivity in their caregiving experiences and self-control during difficult times and stressful events. However, others have reported that different types of coping strategies are needed by the primary caregivers at different times such as seeking support from friends and families, or work satisfaction and comfort food [10]. According to Lara and de lo Pinos [44], primary caregivers with CWD feel greater self-worth and generate superior skills in improving the quality of care for the CWD that may be extended to other children in the family.

As reported by Elsayed and colleagues [45], sleeping problems are more common in children with CP compared to children with normal development. The study reported 46.2% to 62.5% of children with CP experienced sleeping problems such as early insomnia, sleep bruxism, sleep disordered breathing, nightmares, sleep talking and prolonged daytime napping. Sleeping problems do not only affect the health of the child, but are also taxing on the well-being of primary caregivers, as they require primary caregivers’ attention at night. Our current study has identified that sleeping problems among children with CP caused lower HRQOL among their primary caregivers. Similar to this finding, Wayte and colleagues [46] also reported a significant correlation between child and maternal sleep disturbances, which further led to maternal depression.

The coefficient of determinations (*R*^2^) of the final model for Total Family Impact, Parent HRQOL and Family Functioning were relatively low (i.e., between 8.6% and 11.0%). This suggests that the socio-economic demographics of the family and the health condition of the children with CP only affected the well-being of primary caregivers and family functioning to the minimum extent. There is a need to explore other intrinsic factors that contribute to HRQOL and family functioning of primary caregivers of children with CP in Malaysia, such as the child’s attitude and behavior, primary caregivers’ coping mechanisms, social support systems, and psychological well-being.

Generalizability of findings reported in the current study is limited. Even though the studied primary caregivers were recruited from different cities of Malaysia, most of them had their children with CP registered with government- or community-based rehabilitation centers, which provide services predominantly to the low to medium socio-economic status population. There is a need for further research on the Malaysian population of higher socio-economic status and other population that has not registered their children with any type of services for CP. Parenting interventions such as Stepping Stones Triple P (SSTP) that focuses on enhancing parenting skills, have been reported to reduce problem behaviors of CWD [47]. By identifying the challenges faced by primary caregivers of children with CP, Whittingham et al. [15] explored the value of adopting SSTP intervention and had reported significant reductions in stress and depressive symptoms when primary caregivers received a combination of SSTP with Acceptance and Commitment Therapy (ACT) [48]. It would also be of interest to explore the potential benefits of adopting SSTP and ACT approaches to Malaysian primary caregivers of children with CP.

Use of KI sampling method to recruit the respondents instead of random sampling method might also affect the generalizability of the current findings. However, the decision was made upon several considerations. First, the study population is relatively niche and recruitment of primary caregivers with children with CP without referral from local authorities and NGOs are difficult. Second, given that the larger part of this project was to provide free clinical and health services to potential respondents, the KI sampling method could help the research team to effectively pinpoint those respondents who need the services, as the KIs were trained to identify children with severe impairments at the community level. Third, through the KIs, families with especially nonregistered CWD were encouraged to have their children’s health status assessed and for the primary caregivers to gain more information on general healthcare of their children by attending the health screening camps.

## 5. Conclusions

To conclude, the current study offers an insight into the relationship between socio-economic demographics of the family of children with CP, the complication faced by children with CP, primary caregivers’ QOL, and family functioning. In the context of Malaysia, primary caregivers are experiencing decent health-related quality of life and family functioning. Despite that, primary caregivers’ well-being and family functioning should always be regarded as pivotal issue as they affect the overall well-being of family members, as well as the positive growth of children with CP. The current study provides new knowledge that may guide the development of parenting guidelines for primary caregivers and their children with CP. The current research has also highlighted the urgency of exploring other potential elements that could enhance primary caregivers HRQOL and family functioning in Malaysia.

## Figures and Tables

**Table 1:** Profile of the Primary Caregivers (*n* = 159).

Primary Caregivers’ Profile	Frequency (%)	M (SD) ^1^
**Biological sex**		
Male	30 (18.9)	
Female	127 (79.9)	
Missing data	2 (1.3)	
**Relationship to the children**		
Father	29 (18.2)	
Mother	120 (75.5)	
Guardian	10 (6.3)	
**Residing state**		
Kelantan	35 (22.0)	
Johor	81 (50.9)	
Sarawak	43 (27.0)	
**Level of education (Child’s father)**		
No education or primary school	18 (11.3)	
Secondary school	89 (56.0)	
Beyond secondary school	45 (28.3)	
Missing data	7 (4.4)	
**Level of education (Child’s mother)**		
No education or primary school	22 (13.8)	
Secondary school	84 (52.8)	
Beyond secondary school	51 (32.1)	
Missing data	2 (1.3)	
**Family monthly income**		
Less than RM2000 (~USD494)	69 (43.4)	
RM2000-RM3999 (~USD494–988)	46 (28.9)	
RM4000 (~USD988) and more	44 (27.7)	
**Children with other disability**		
Yes	15 (9.4)	
No	141 (88.7)	
Missing data	3 (1.9)	
**Age**		42.8 (8.4)

^1^ M = Mean; SD = Standard deviation.

**Table 2:** Profile of the children with CP (*n* = 159).

Children’s Profile	Frequency (%)	M (SD) ^1^
**Biological sex**		
Male	94 (59.1)	
Female	65 (40.9)	
**Age group**		
Less than 2 years old	3 (1.9)	
2–4 years old	23 (14.5)	
5–7 years old	25 (15.7)	
8–12 years old	50 (31.4)	
13–18 years old	58 (36.5)	
**Attending school**		
Yes	78 (49.1)	
No	79 (49.7)	
Missing data	2 (1.3)	
**Access to health care Services** (Multiple Choice)		
Hospital and clinic	99 (62.3)	
Rehabilitation center	89 (56.0)	
**Types of CP**		
Spastic	124 (78.0)	
Ataxic	11 (6.9)	
Choreoathetoid	5 (3.1)	
Mixed	17 (10.7)	
Missing data	2 (1.3)	
**Severity of CP (based on GMFCS ^2^)**		
I	21 (13.2)	
II	21 (13.2)	
III	18 (11.3)	
IV	34 (21.4)	
V	62 (38.4)	
None	4 (2.5)	
**Other Comorbidities (multiple choice)**		
Sleeping problems	30 (18.9)	
Hearing problems	13 (8.2)	
Behavior problems	25 (15.7)	
Epilepsy	58 (36.5)	
Eye cortical blindness/squint	46 (28.9)	
Speech problems	105 (66.0)	
Swallowing/chewing problems	32 (20.1)	
Salivation	48 (30.2)	
Gastroesophageal reflux disease	11 (6.9)	
Hyperactive airway diseases	4 (2.5)	
Constipation	45 (28.3)	
Bowel/Urinary problems	81 (50.9)	
Hip dislocation	18 (11.3)	
Learning disability	90 (56.6)	
**Numbers of Comorbidities**		4.0 (3.0)

^1^ M = Mean; SD = Standard deviation. ^2^ The Gross Motor Function Classification System (GMFCS) objectively classifies one’s gross motor function into 5 levels, based on sitting and walking ability, and wheeled mobility. It has proven to be reliable in distinguishing gross motor function, high accuracy in predicting future motor function, and stable over time [21].

**Table 3:** Mean Score and Cronbach’s Alpha of PedsQL Family Impact Module Among Participants (*n* = 159).

Domain in Family Impact Module	No. of Items	M ^1^	SD ^2^	α ^3^
Physical functioning	6	78.6	17.19	0.803
Emotion functioning	5	82.4	16.59	0.805
Social functioning	4	84.6	17.72	0.716
Cognitive functioning	5	85.0	16.55	0.830
Communication	3	83.9	18.20	0.672
Worry	5	80.7	18.47	0.671
Daily activities	3	76.1	23.84	0.840
Family relationship	5	90.9	13.81	0.825
Parent HRQOL Summary Score	20	82.4	13.64	0.903
Family Functioning Summary Score	8	85.3	14.69	0.823
Total Impact Score	36	81.9	12.90	0.934

^1^ M = Mean; ^2^ SD = Standard deviation; ^3^ α = Cronbach’s alpha. According to the general guideline for Cronbach’s alpha, a coefficient value of 0.7 and above indicates adequate reliability but 0.65 is acceptable [22,23].

**Table 4:** Factors Associated with Total Family Impact (*n* = 159).

Predictors	Simple Linear Regression		Multiple Linear Regression	
	b * (95% CI)	*p*	B # (95% CI)	*p*
**Mother’s level of education**				
No education/primary	−0.24 (−6.31, 5.84)	0.939	0.68 (−5.32, 6.67)	0.824
Secondary (Ref.)	-	-	-	-
Beyond secondary	3.94 (−0.57, 8.44)	0.086	8.70 (3.42, 13.97)	0.001
**Family monthly income**				
Low (Ref.)	-	-	-	-
Medium	−2.69 (−7.53, 2.15)	0.274	−3.48 (−8.44, 1.48)	0.186
High	−3.58 (−8.49, 1.33)	0.151	−8.82 (−14.63, −3.02)	0.003

Dependent variable for both regressions was Total impact score. Backward method was applied. The model reasonably fits. There were no multicollinearity problems and interactions detected. Model assumptions of linearity, independent sample, normality, and homoscedasticity were fulfilled. Coefficient of determination (*R*^2^) is 0.110. * Crude regression coefficient. # Adjusted regression coefficient. Ref. = Reference category.

**Table 5:** Factors Associated with Primary Caregiver’s Health-Related Quality of Life (*n* = 159).

Predictors	Simple Linear Regression		Multiple Linear Regression	
	b * (95% CI)	*p*	b # (95% CI)	*p*
**Mother’s level of education**				
No education/primary	−2.01 (−8.44, 4.43)	0.539	−1.54 (−7.88, 4.79)	0.631
Secondary (Ref.)	-	-	-	-
Beyond secondary	3.55 (−1.22, 8.32)	0.143	7.01 (1.55, 12.48)	0.012
**Family monthly income**				
Low (Ref.)	-	-	-	
Medium	−3.61 (−8.73, 1.52)	0.166	−4.97 (−10.17, 0.23)	0.061
High	−3.04 (−8.24, 2.15)	0.249	−7.64 (−13.66, −1.62)	0.013
**Types of comorbidities**				
Sleeping problems	−5.41 (−10.83, −0.00)	0.050	−5.93 (−11.31, −0.55)	0.031

Dependent variable for both regressions was Parent Summary HRQOL Score. Backward method applied. The model reasonably fits. There were no multicollinearity problems and interactions detected. Model assumptions of linearity, independent sample, normality, and homoscedasticity were fulfilled. Coefficient of determination (*R*^2^) is 0.086. * Crude regression coefficient. # Adjusted regression coefficient. Ref. = Reference category.

**Table 6:** Factors Associated with Family Functioning (*n* = 159).

Predictors	Simple Linear Regression		Multiple Linear Regression	
	b * (95% CI)	*p*	b # (95% CI)	*p*
**Mother’s Education**				
No Education/Primary	−0.65 (−7.59, 6.29)	0.854	0.77 (−6.03, 7.58)	0.822
Secondary (Ref.)	-	-	-	-
Beyond Secondary	3.72 (−1.43, 8.86)	0.156	9.20 (3.21, 15.20)	0.003
**Family Monthly Income**				
Low (Ref.)	-	-	-	
Medium	−1.87 (−7.39, 3.66)	0.505	−2.00 (−7.64, 3.64)	0.485
High	−4.03 (−9.63, 1.56)	0.156	−8.99 (−15.56, −2.43)	0.008
**Children with Other Disability**	7.91 (0.06, 15.75)	0.048	11.83 (3.71, 19.95)	0.005

Dependent variable for both regressions was Family Functioning Summary Score. Backward method applied. The model reasonably fits. There were no multicollinearity problems and interactions detected. Model assumptions of linearity, independent sample, normality, and homoscedasticity were fulfilled. Coefficient of determination (*R*^2^) is 0.103. * Crude regression coefficient. # Adjusted regression coefficient. Ref. = Reference category.

## Data Availability

The data are available on request from the corresponding authors.

## References

[1] Department of Statistics Malaysia (2019). Children Statistics, Malaysia. https://www.dosm.gov.my/v1/index.php?r=column/ctwoByCat&parent_id=123&menu_id=U3VPMldoYUxzVzFaYmNkWXZteGduZz09#.

[2] Gan Z. (2019). Issue Brief: Children with Disabilities in Malaysia. https://www.unicef.org/malaysia/reports/issue-brief-children-disabilities-malaysia.

[3] Thompson S., Disability Prevalence and Trends (2017). K4D Helpdesk Report. https://gsdrc.org/publications/disability-prevalence-and-trends/.

[4] Abdullah N., Hanafi H., Hamdi N. (2017). The rights of persons with disabilities in Malaysia: The underlying reasons for ineffectiveness of Persons with Disabilities Act 2008. Int. J. Stud. Child. Women Elder. Disabil..

[5] United Nations Children’s Fund (UNICEF) Malaysia (2014). Children with Disabilities in Malaysia: Mapping the Policies, Programmes, Interventions and Stakeholders. https://www.unicef.org/malaysia/UNICEF-Children_with_Disability_in_Malaysia_2014_lowres.pdf.

[6] Aneja S. (2004). Evaluation of a child with cerebral palsy. Indian J. Pediatr..

[7] Oskoui M., Coutinho F., Dykeman J., Jetté N., Pringsheim T. (2013). An update on the prevalence of cerebral palsy: A systematic review and meta-analysis. Dev. Med. Child Neurol..

[8] Schiariti V., Selb M., Cieza A., O’donnell M. (2015). International classification of functioning, disability and health core sets for children and youth with cerebral palsy: A consensus meeting. Dev. Med. Child Neurol..

[9] Winter S., Autry A., Boyle C., Yeargin-Allsopp M. (2002). Trends in the prevalence of cerebral palsy in a population-based study. Pediatrics.

[10] Whittingham K., Wee D., Sanders M.R., Boyd R. (2013). Sorrow, coping and resiliency: Parents of children with cerebral palsy share their experiences. Disabil. Rehabil..

[11] Eddy L.L., Engel J.M. (2008). The impact of child disability type on the family. Rehabil. Nurs..

[12] Harun D., Yew E., Ahmad M., Baharudin N.S. (2017). Coping skills and psychosocial adjustments among parents of children with learning disabilities (LD). ASEAN J. Psychiatry.

[13] Isa S.N.I., Ishak I., Ab Rahman A., Saat N.Z.M., Din N.C., Lubis S.H., Ismail M.F.M. (2017). Perceived stress and coping styles among Malay caregivers of children with learning disabilities in Kelantan. Malays. J. Med. Sci..

[14] Carlsson M., Olsson I., Hagberg G., Beckung E. (2008). Behaviour in children with cerebral palsy with and without epilepsy. Dev. Med. Child Neurol..

[15] Whittingham K., Wee D., Sanders M., Boyd R. (2011). Responding to the challenges of parenting a child with cerebral palsy: A focus group. Disabil. Rehabil..

[16] Barlow J.H., Cullen-Powell L.A., Cheshire A. (2006). Psychological well-being among mothers of children with cerebral palsy. Early Child Dev. Care.

[17] Isa S.N.I., Aziz A.A., Ab Rahman A., Ibrahim M.I., Ibrahim W.P.W., Mohamad N., Othman A., Rahman N.A., Harith S., Van Rostenberghe H. (2013). The impact of children with disabilities on parent health-related quality of life and family functioning in Kelantan and its associated factors. J. Dev. Behav. Pediatr..

[18] Hawthorne G., Herrman H., Murphy B. (2006). Interpreting the WHOQOL-BREF: Preliminary population norms and effect sizes. Soc. Indic. Res..

[19] Varni J.W., Sherman S.A., Burwinkle T.M., Dickinson P.E., Dixon P. (2004). The PedsQL™ family impact module: Preliminary reliability and validity. Health Qual. Life Outcomes.

[20] Isa S.N.I., Ishak I., Ab Rahman A., Saat N.Z.M., Din N.C., Lubis S.H., Ismail M.F.M., Suradi N.R.M. (2018). A psychometric evaluation of the Malay version of PedsQL^TM^ Family Impact Module among caregivers of children with learning disabilities. ICGH Conference Proceedings, Proceedings of the 1st International Conference on Global Health, JS Luwansa Hotel, Indonesia, 9–11 November 2016.

[21] Rosenbaum P., Eliasson A.C., Hidecker M.J.C., Palisano R.J. (2014). Classification in childhood disability: Focusing on function in the 21st century. J. Child Neurol..

[22] Taber K.S. (2018). The use of Cronbach’s alpha when developing and reporting research instruments in science education. Res. Sci. Educ..

[23] van Griethuijsen R.A., van Eijck M.W., Haste H., den Brok P.J., Skinner N.C., Mansour N., Gencer A.S., BouJaoude S. (2015). Global patterns in students’ views of science and interest in science. Res. Sci. Educ..

[24] Dambi J.M., Jelsma J., Mlambo T. (2015). Caring for a child with cerebral palsy: The experience of Zimbabwean mothers. Afr. J. Disabil..

[25] Parisi L., Ruberto M., Precenzano F., Di Filippo T., Russotto C., Maltese A., Salerno M., Roccella M. (2016). The quality of life in children with cerebral palsy. Acta Med. Mediterr..

[26] Raina P., O’Donnell M., Rosenbaum P., Brehaut J., Walter S.D., Russell D., Swinton M., Zhu B., Wood E. (2005). The health and well-being of caregivers of children with cerebral palsy. Pediatrics.

[27] Sawyer M., Bittman M., La Greca A., Crettenden A., Borojevic N., Raghavendra P., Russo R. (2011). Time demands of caring for children with cerebral palsy: What are the implications for maternal health?. Dev. Med. Child Neurol..

[28] Böling S., Varho T., Kiviranta T., Haataja L. (2016). Quality of life of Finnish children with cerebral palsy. Disabil. Rehabil..

[29] Dehghan L., Dalvand H., Feizi A., Samadi S.A., Hosseini S.A. (2016). Quality of life in mothers of children with cerebral palsy: The role of children’s gross motor function. J. Child Health Care.

[30] Surender S., Gowda V.K., Sanjay K.S., Basavaraja G.V., Benakappa N., Benakappa A. (2016). Caregiver-reported health-related quality of life of children with cerebral palsy and their families and its association with gross motor function: A South Indian study. J. Neurosci. Rural Pract..

[31] Pretorius C., Steadman J. (2018). Barriers and facilitators to caring for a child with cerebral palsy in rural communities of the Western Cape, South Africa. Child Care Pract..

[32] Benfer K.A., Jordan R., Bandaranayake S., Finn C., Ware R.S., Boyd R.N. (2014). Motor severity in children with cerebral palsy studied in a high-resource and low-resource country. Pediatrics.

[33] Glinac A., Matović L., Delalić A., Mešalić L. (2017). Quality of life in mothers of children with cerebral palsy. Acta Clin. Croat..

[34] Kołtuniuk A., Rozensztrauch A., Budzińska P., Rosińczuk J. (2019). The quality of life of Polish children with cerebral palsy and the impact of the disease on the family functioning. J. Pediatr. Nurs..

[35] Zuurmond M., O’Banion D., Gladstone M., Carsamar S., Kerac M., Baltussen M., Tann C.J., Gyamah Nyante G., Polack S. (2018). Evaluating the impact of a community-based parent training programme for children with cerebral palsy in Ghana. PLoS ONE.

[36] Bella G.P., Garcia M.C., Spadari-Bratfisch R.C. (2011). Salivary cortisol, stress, and health in primary caregivers (mothers) of children with cerebral palsy. Psychoneuroendocrinology.

[37] Rudd R.E. (2007). Health literacy skills of US adults. Am. J. Health Behav..

[38] Kickbusch I., Pelikan J.M., Apfel F., Tsouros A.D. (2013). Health Literacy. The Solid Facts.

[39] Khayatzadeh M.M., Rostami H.R., Amirsalari S., Karimloo M. (2013). Investigation of quality of life in mothers of children with cerebral palsy in Iran: Association with socio-economic status, marital satisfaction and fatigue. Disabil. Rehabil..

[40] Basri N.A., Hashim N.N.W.N. (2019). Stress in parents of children with Autism: A Malaysian experience. Intellect. Discourse.

[41] Mohamad M.S., Subhi N., Zakaria E., Aun N.S.M. (2013). Cultural influences in mental health help-seeking among Malaysian family caregivers. Pertanika J. Soc. Sci. Hum..

[42] Haque A. (2005). Mental health concepts and program development in Malaysia. J. Ment. Health.

[43] Ilias K., Liaw J.H.J., Cornish K., Park M.S.A., Golden K.J. (2017). Wellbeing of mothers of children with “AUTISM” in Malaysia: An interpretative phenomenological analysis study. J. Intellect. Dev. Disabil..

[44] Lara E.B., de los Pinos C.C. (2017). Families with a disabled member: Impact and family education. Procedia Soc. Behav. Sci..

[45] Elsayed R.M., Hasanein B.M., Sayyah H.E., El-Auoty M.M., Tharwat N., Belal T.M. (2013). Sleep assessment of children with cerebral palsy: Using validated sleep questionnaire. Ann. Indian Acad. Neurol..

[46] Wayte S., McCaughey E., Holley S., Annaz D., Hill C.M. (2012). Sleep problems in children with cerebral palsy and their relationship with maternal sleep and depression. Acta Paediatr..

[47] Roberts C., Mazzucchelli T., Studman L., Sanders M.R. (2006). Behavioral family intervention for children with developmental disabilities and behavioral problems. J. Clin. Child Adolesc. Psychol..

[48] Whittingham K., Sanders M.R., McKinlay L., Boyd R.N. (2016). Parenting intervention combined with acceptance and commitment therapy: A trial with families of children with cerebral palsy. J. Pediatr. Psychol..

